# Dual-Frequency Doppler LiDAR Based on External Optical Feedback Effect in a Laser

**DOI:** 10.3390/s20216303

**Published:** 2020-11-05

**Authors:** Zhuqiu Chen, Yanguang Yu, Yuxi Ruan, Bairun Nie, Jiangtao Xi, Qinghua Guo, Jun Tong

**Affiliations:** School of Electrical, Computer and Telecommunications Engineering, University of Wollongong, Northfields Avenue, Wollongong 2522, NSW, Australia; zc606@uowmail.edu.au (Z.C.); yr776@uowmail.edu.au (Y.R.); bn807@uowmail.edu.au (B.N.); jiangtao@uow.edu.au (J.X.); qguo@uow.edu.au (Q.G.); jtong@uow.edu.au (J.T.)

**Keywords:** doppler LiDAR, dual-frequency laser, optical feedback, velocity measurement, laser dynamics

## Abstract

A novel Dual-frequency Doppler LiDAR (DFDL) is presented where the dual-frequency light source is generated by using external optical feedback (EOF) effect in a laser diode (LD). By operating a LD at period-one (P1) state and choosing suitable LD related parameters, a dual-frequency light source can be achieved. Such a dual-frequency source has advantages of the minimum part-count scheme, low cost in implementation, and ease in optical alignment. Theory and system design are presented for the proposed DFDL for velocity measurement with high measurement resolution. The proposed design has a potential contribution to the Light Detection And Ranging (LiDAR) in practical engineering applications.

## 1. Introduction

Light Detection And Ranging (LiDAR) is a method for measuring distances (ranging) by illuminating the target with laser light and measuring the reflection with a sensor [1]. LiDAR systems have been widely investigated and used since the 1970s. Its advantages such as good directionality, high spatial resolution, and noninvasive in velocity detection have enabled LiDAR to be extensively employed in numerous applications [2]. In remote sensing, LiDARs are coupled with visual sensors to build highly robust scan-matching approaches aiming to achieve simultaneous localization and mapping (SLAM). LiDARs are able to provide high resolution when assessing building damages caused by earthquakes. Wind LiDARs are used in measurements of the atmospheric turbulence. In modeling and imaging, LiDAR applications include scene reconstruction and terrain modeling with high quality utilizing cameras-LiDARs fusion in planetary and terrestrial robotics [3,4,5,6]. In the early stage of LiDAR systems, the backscattered single frequency laser light reflected by the moving target and would utilize the Doppler effect. The velocity information can be extracted from the Doppler shift frequency through signal processing technology [7,8,9]. The single frequency Doppler LiDAR systems have the ability to measure wind velocities with a deviation over ranges of up to 0.39 m/s [10], to achieve the atmospheric measurement over a 51-day continuous and unattended field deployment with a range of 7.5 km for observing the boundary layer [11], and detect air turbulence in clear air at a range of 9.3 km at cruising altitudes [12]. However, they are highly sensitive to external disturbances. For example, the spectral bandwidth of the Doppler-shifted line could be broadened by speckle noise and optical noise caused by the roughness of the target and the coherence properties of a laser. Consequently, the velocity measurement resolution will be degraded. To overcome these disadvantages, an alternative solution known as Dual-frequency Doppler LiDAR (DFDL) has emerged. This approach utilizes a microwave beat frequency which contains two optical frequency components [13,14,15,16]. The velocity of a moving target can be measured by identifying the difference of doppler frequency shifts of two frequency components. Currently, the generation of dual-frequency can be achieved in a number of ways, e.g., utilizing dual-frequency laser that emits dual-frequency laser beam by inserting two quarter-wave plates inside the laser cavity; Combining two single-frequency laser beams to create a dual-frequency laser beam; Injecting two single-frequency beams from two master lasers into one slave laser; Using one pair of master-slave lasers, where the slave laser is operating in period-one oscillation state to emit dual-frequency [15,17,18,19,20]. However, these methods require two or more laser diodes (LDs) for generating dual-frequency. The method in [19] requires only one LD but an expensive frequency shifter (acousto-optic modulator) is needed. These requirements lead to a complicated and expensive system.

In this paper, we proposed a new method to generate a dual-frequency source by using laser dynamics. Laser diodes (LD) with external optical feedback (EOF) are known to demonstrate complex dynamics, which may give rise to negative effects on the LD performance, e.g., degrading the modulation response characteristics, enhancing laser intensity noise, etc [21]. Meanwhile, such EOF effect in a LD also enables many applications, e.g., a class of laser interferometry, termed optical feedback interferometry, or self-mixing interferometry (SMI). As a promising non-contact sensing technology, EOF has attracted intensive research in recent decades due to the merits of the minimum part-count scheme, low cost in implementation, and ease in optical alignment [22,23,24,25,26]. With the increase of the optical feedback, a LD will leave the steady state and enter other dynamic states such as period-one (P1) oscillation, multi-periodic oscillation, and chaos, and rich dynamics can then be observed [27]. In recent years, LD dynamics have been investigated and found their various potential applications in high-resolution sensing, photonic microwave generation, balance detection, chaotic radar, etc. [28,29,30,31,32,33]. In this paper, a novel DFDL system is proposed. The dual-frequency is generated by using a LD with EOF operating at P1 state. The related theory design and analysis are presented. The results show that the proposed DFDL system can reach up to 4.8 μm/s velocity measurement resolution with 31.21 GHz microwave beat frequency.

## 2. Generation of Dual-Frequency Laser

**Figure 1:**
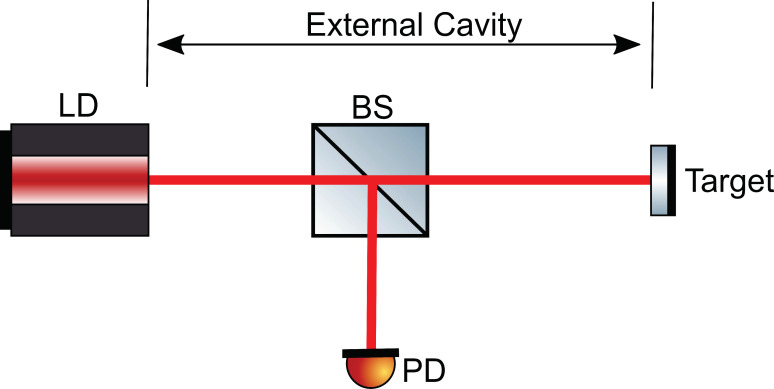
Schematic diagram of a laser diode (LD) with the external optical feedback (EOF) system.

Laser dynamics induced by EOF is studied by using the set up shown in Figure 1. It consists of a LD and an external target. The length between the LD front facet and the external target is defined as the external cavity length denoted by L. The laser intensity is captured by an external photodiode (PD).

The dynamics of an EOF system can be described by the well-known Lang and Kobayashi (L-K) equations, as shown in Equations (1)–(3) [34]. In which E(t) is the amplitude of the electric field, ϕ(t) is the electric field phase, and N(t) is the carrier density.

(1) $$\frac{dE(t)}{dt}=\frac{1}{2}\left\{G\left[N(t),E(t)\right]-\frac{1}{\tau_{p}}\right\}E(t)+\frac{\kappa }{\tau_{in}}\cdot E(t-\tau )\cdot \cos\left[\omega_{0}\tau +\phi (t)-\phi (t-\tau )\right]$$

(2) $$\frac{d\phi (t)}{dt}=\frac{1}{2}\alpha \left\{G\left[N(t),E(t)\right]-\frac{1}{\tau_{p}}\right\}-\frac{\kappa }{\tau_{in}}\cdot \frac{E(t-\tau )}{E(t)}\cdot \sin\left[\omega_{0}\tau +\phi (t)-\phi (t-\tau )\right]$$

(3) $$\frac{dN(t)}{dt}=\frac{J}{eV}-\frac{N(t)}{\tau_{s}}-G\left[N(t),E(t)\right]E^{2}(t)$$

where $G\left[N(t),E(t)\right]=G_{N}[N(t)-N_{0}][1-\varepsilon \Gamma E^{2}(t)]$ is the modal gain per unit time. Physical meanings and values of parameters used in Equations (1)–(3) can be found in Table 1.

The parameters shown in Table 1 can be classified as LD internal parameters that are fixed values for a given LD, and controllable parameters associated with the external cavity and LD states. The laser intensity $E^{2}(t)$ can be obtained through numerical solving the L-K equations. The LD dynamics states can be identified by observing the waveform of $E^{2}(t)$.

**Figure 2:**
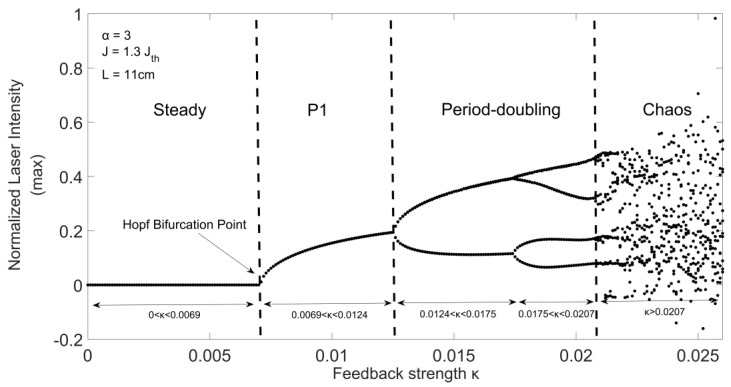
Bifurcation diagram for a LD with EOF system.

Through changing one or more controllable parameters, a LD may experience different dynamic states, including steady state, P1 oscillation state, period-doubling oscillation state, and chaos. The routing from steady state to chaos when a specific parameter change is called bifurcation. To generate a bifurcation diagram, the local maximum (or minimum) of the waveform of $E^{2}(t)$ denoted by $E_{\max}{}^{2}(t)$ are sampled for a set parameters given for a LD and its external cavity. As an example, Figure 2 presents a bifurcation diagram with parameters: α = 3, L = 11 cm and $J\;=\;1.3J_{th}$ ($J_{th}$ is the LD current threshold), other parameter values refer to Table 1. As we are interested in how the EOF strength κ influences the LD states. Hence, we vary κ from 0 to 0.025 with a step size of 0.0001. At each feedback strength κ, we obtained the corresponding LD state and present it on the bifurcation diagram in a 2-D plane ($E_{\max}{}^{2}(t)$, κ) on Figure 2, different states are indicated with corresponding κ ranges.

It can be seen from Figure 2, the steady state corresponds to the range of κ with 0<κ<0.0069. With the further increase of κ, undamped relaxation oscillation will occur after the Hopf bifurcation point, which causes the system to enter the P1 oscillation state region with 0.0069<κ<0.0124, and routes to period-2 (P2) state with 0.0124<κ<0.0175, period-4 (P4) state with 0.0175<κ<0.0207. Eventually, due to the large κ, the system collapsed and presents chaos state when κ>0.0207. Generally, P2 and P4 states can also be collectively referred to as period-doubling.

**Figure 3:**
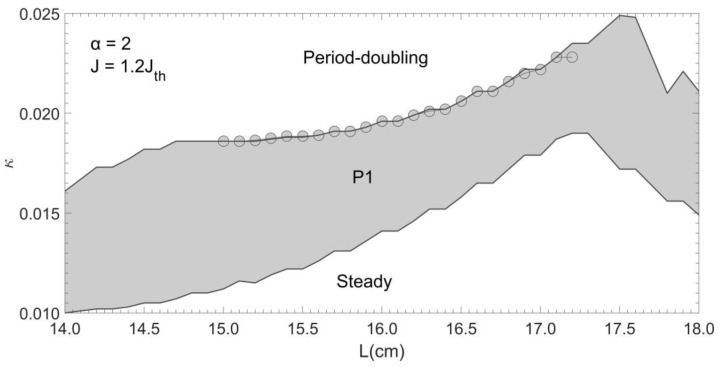
State diagram under the parameter space (κ, L).

Next, let us make an investigation on how to generate a dual-frequency laser signal at P1 state. Both feedback strength κ and external cavity length L are varying and are treated as control parameters for LD state. The cavity length L starts from 14 cm to 18 cm with a step of 0.1 cm. For each L, κ increase from 0 to 0.025 with a step size of 0.0001. By observing the waveform of $E^{2}(t)$, the corresponding LD states are recorded and indicated on the (κ , L) plane shown in Figure 3.

**Figure 4:**
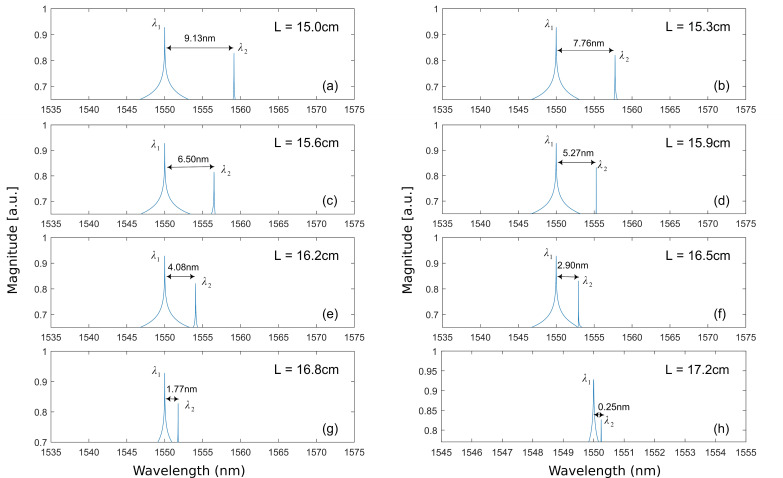
Optical spectrum of the dual-frequency laser signals. (**a**–**h**) with different external cavity length.

The circles shown in Figure 3 indicate the corresponded (κ , L) values with which the LD is able to generate a dual-frequency laser signal. We found the region that can generate dual-frequency to be only within L=15.0 cm to L=17.2 cm and are on the boundary between the P1 region and the period-doubling region. The dual-frequency spectrum will disappear once the external cavity length is beyond the range. The wavelength difference of the two frequency is denoted as $∆\lambda =\left|\lambda_{2}-\lambda_{1}\right|$, where $\lambda_{1}$ and $\lambda_{2}$ are two corresponding wavelengths of two frequency components $f_{1}$ and $f_{2}$. Its corresponding frequency is known as beat frequency $f_{beat}=\left|f_{2}-f_{1}\right|=\left|c/\lambda_{2}-c/\lambda_{1}\right|$. Figure 4 shows eight examples of optical spectrum for dual-frequency laser signals generated for certain (κ , L) parameter pairs. Figure 4a shows the generated two wavelengths are $\lambda_{1}\;=\;1550.00\;\mathrm{nm}$ and $\lambda_{2}\;=\;1559.13\;\mathrm{nm}$ when L = 15.0 cm, which cause a ∆λ = 9.13 nm. This results a beat frequency $f_{beat}=1133.40\;\mathrm{GHz}$. From Figure 4b–h, we further increase L from 15.1 cm to 17.2 cm, the corresponding wavelength difference ∆λ decrease from 8.46 nm to 0.24 nm.

**Figure 5:**
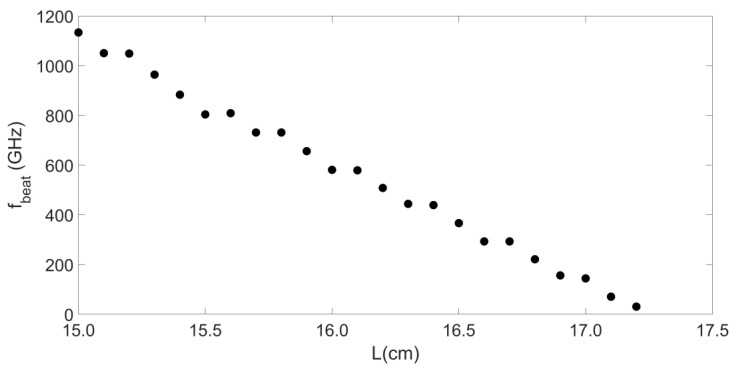
Relationship between $f_{beat}$ and L.

We also established the relationship between the $f_{beat}$ and L shown in Figure 5. It shows the $f_{beat}$ changes from 1050.40 GHz to 31.21 GHz with increasing L from 15.1 cm to 17.2 cm. These results show that a tunable microwave frequency can be generated by varying the external length L, which provides flexibility for generating a desired dual-frequency laser signal for different application needs. Besides, results also indicate that it is not a perfect linear decline process. After a period of decline, two consecutives $f_{beat}$ values will be the same. This shows that when a specific $f_{beat}$ is generated in this way, it is robust to adjust the length of the external length L when J and α are fixed.

## 3. Setup and Principle of DFDL System

**Figure 6:**
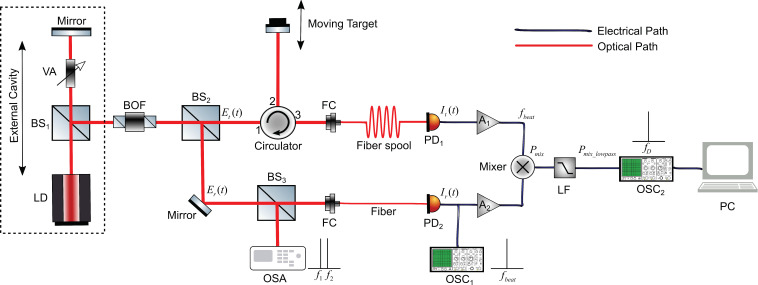
Experimental setup of Dual-frequency Doppler LiDAR (DFDL) system. LD, single-mode laser diode; MFS, microwave frequency synthesizer; BS, beam splitter; VA, variable attenuator; BOF, bandpass optical filter; FC, fiber coupler; Circulator; Fiber and Fiber spool; ${\mathrm{PD}}_{1}$ and ${\mathrm{PD}}_{2}$, high-speed photodiodes; $A_{1}$ and $A_{2}$, microwave amplifiers; Mixer, microwave mixer; ${\mathrm{OSC}}_{1}$ and ${\mathrm{OSC}}_{2}$, digital oscilloscopes; OSA, optical spectrum analyzer; LF, lowpass filter; PC, computer.

In Section 2, we have demonstrated that an EOF system operating in P1 state can generate a dual-frequency signal. In this section, we utilized this feature and use it as the dual-frequency light source in the DFDL system. The schematic layout of the DFDL system is depicted in Figure 6. The boxed area is an EOF system that plays a role as the dual-frequency light source, it consists of a LD, a mirror, and a variable attenuator (VA). By varying the VA to adjust the optical feedback strength, thus, the LD can operate in P1 state to emit dual-frequency light.

A fraction of the laser beam from the LD is divided by beam splitter ${\mathrm{BS}}_{1}$, then directed to a bandpass optical filter (BOF), this is to reduce the sidebands and noise in the dual-frequency light. Then the light is split into two beams again by ${\mathrm{BS}}_{2}$, one beam named target measurement beam with the electric field $E_{t}(t)$ is sent to the port 1 of a circulator and comes out from port 2, then it is reflected by a target moving with a constant speed v. The moving target will introduce the Doppler effect and results in a frequency shift of the generated beat frequency signal $f_{beat}$ denoted as $f_{D}=|f_{D2}-f_{D1}|$, where $f_{D1}$ and $f_{D2}$ are Doppler shift frequency of $f_{1}$ and $f_{2}$, respectively. $f_{D}$ is also called Doppler shift frequency difference. Then, the light with the electric field $E_{t}(t)$ comes out from port 3 and is coupled into a long length fiber spool (in tens of km). ${\mathrm{PD}}_{1}$ transfers electric field to photocurrent denoted as $I_{t}(t)$. Another beam from ${\mathrm{BS}}_{2}$ named as reference beam with the electric field $E_{r}(t)$ is then split again by ${\mathrm{BS}}_{3}$ and directed to an optical spectrum analyzer (OSA) and ${\mathrm{PD}}_{2}$ respectively. The OSA is used to visualize the dual-frequency laser spectrum (with components $f_{1}\;\mathrm{and}\;f_{2}$). The output photocurrent of ${\mathrm{PD}}_{2}$ with the frequency $f_{beat}$ is denoted as $I_{r}(t)$ and its electrical spectrum can be recorded by the oscilloscope ${\mathrm{OSC}}_{1}$ to get $f_{beat}$. Both photocurrents $I_{t}(t)$ and $I_{r}(t)$ from ${\mathrm{PD}}_{1}$ and ${\mathrm{PD}}_{2}$ are boosted by microwave amplifiers denoted by $A_{1}$ and $A_{2}$ with gain factor $M_{1}$ and $M_{2}$, and then mixed inside a microwave mixer. The output of the mixer is sent to the ${\mathrm{OSC}}_{2}$ to acquire the Doppler signal and recorded on a PC, from which $f_{D}$ can be measured.

The dual-frequency light containing two optical frequencies components ($f_{1}$ and $f_{2}$) are with light magnitude as $E_{1}$ and $E_{2}$, respectively. For the reference beam, the electric field of the dual-frequency signal can be expressed as,

(4) $$E_{r}(t)=E_{1}e^{i[\phi_{1}(t)-2\pi f_{1}t]}+E_{2}e^{i[\phi_{2}(t)-2\pi f_{2}t]}$$

where $\phi_{1}(t)$ and $\phi_{2}(t)$ are the two optical phases relating to the two frequency components $f_{1}$ and $f_{2}$, respectively. Then, for the target measurement beam, the signal received at the ${\mathrm{PD}}_{1}$ can be expressed as below, a delay τ (round trip) is caused due to the moving target. This is written as,

(5) $$E_{t}(t)=E_{1}e^{i[\phi_{1}(t-\tau )-2\pi f_{1}(t-\tau )]}+E_{2}e^{i[\phi_{2}(t-\tau )-2\pi f_{2}(t-\tau )]}$$

where τ=2p/c is the round-trip delay time, p=d+vt is the target position, d is the initial distance of the target, v is the speed of the moving target. The detected current signals from ${\mathrm{PD}}_{1}$ and ${\mathrm{PD}}_{2}$, respectively, are expressed as,

(6) $$I_{t}(t)=2M_{1}E_{1}E_{2}\cos[2\pi f_{beat}t-4p\pi f_{beat}/c-∆\phi (t-\tau )]$$

(7) $$I_{r}(t)=2M_{2}E_{1}E_{2}\cos[2\pi f_{beat}t-∆\phi (t)]$$

where $M_{1}$ and $M_{2}$ are the amplifier gain for the target beam applied by microwave amplifier A_1_ and A_2_, respectively, $∆\phi (t)=\phi_{2}(t)-\phi_{1}(t)$. After passing A_1_ and A_2_, two current signals are sent to a microwave mixer and outputting a signal $P_{mix}$, which is proportional to the product of $I_{r}(t)$ and $I_{t}(t)$. According to Equations (6) and (7), the product of $I_{r}(t)$ and $I_{t}(t)$ is shown below,

(8) $$\begin{array}{cc} P_{mix}= & I_{r}(t)*I_{t}(t)\\ = & 2M_{1}M_{2}E_{1}^{2}E_{2}^{2}\cos(2\pi f_{D}t+4\pi df_{beat}/c-\Phi )+\\ & 2M_{1}M_{2}E_{1}^{2}E_{2}^{2}\cos[2\pi (2f_{beat}-f_{D})t-4\pi df_{beat}/c-∆\phi (t)-∆\phi (t-\tau )] \end{array}$$

where Φ=∆ϕ(t)−∆ϕ(t−τ). It can be seen, the first term in Equation (8) is with low frequency (with frequency $f_{D}$ dominated) and the second term is with very high frequency dominated by ($2f_{beat}-f_{D}$) in GHz. If we make use of the low-frequency component contained in the signal $P_{mix}$, after a lowpass filter processing, we have,

(9) $$P_{mix\_lowpass}=2M_{1}M_{2}E_{1}^{2}E_{2}^{2}\cos(2\pi f_{D}t+4\pi df_{beat}/c-\Phi )$$

$f_{D}$ can be obtained by taking the power spectral density (PSD) on the signal $P_{mix\_lowpass}$. Then the velocity of the moving target can be calculated by,

(10) $$v=cf_{D}/2f_{beat}$$

**Figure 7:**
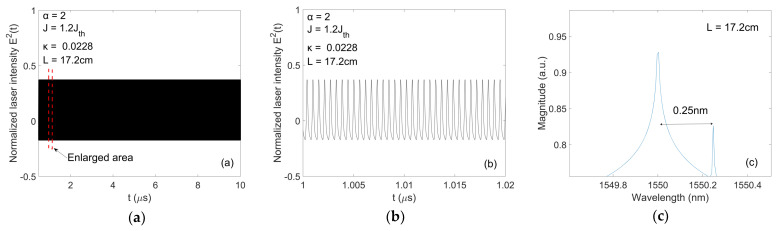
(**a**) LD operates at P1 state; (**b**) Enlargement of boxed area in (**a**); (**c**) Optical spectrum of laser light with two frequency components.

Regarding $f_{beat}$, it is determined by the dual-frequency source system demonstrated in Section 2. As an example, with the parameters setting for the LD given in Figure 4h, $\alpha \;=\;2,\;J\;=\;1.2J_{th},\;L\;=\;17.2\;\mathrm{cm}$, and κ = 0.0228, the system is able to generate a dual-frequency light shown in Figure 7. Figure 7a is the laser intensity ($E^{2}(t)$) waveform. Figure 7b shows an enlargement region in Figure 7a for the time duration from 1 μs to 1.02 μs. Figure 7c shows the optical spectrum containing two wavelengths with $\lambda_{1}\;=\;1550.00\;\mathrm{nm}\;,\lambda_{2}\;=\;1550.25\;\mathrm{nm}$. The wavelength difference is ∆λ=0.25 nm. Thus, the beat frequency $f_{beat}$ is 31.21 GHz. The velocity measurement resolution corresponds to the measurement resolution of $f_{D}$. For instance, if we use Tektronix RSA7100B spectrum analyzer to measure $f_{D}$, the frequency measurement resolution will be $1\;\times \;10^{-3}\;\;\mathrm{Hz}$. This will work out a corresponding velocity resolution as 4.8 μm/s. In Table 2, we show a comparison of our proposed DFDL to some of other dual-frequency methods, it is evident that our proposed DFDL provided a great improvement on velocity measurement resolution.

## 4. Conclusions

With the change of one or more controllable system parameters in EOF system, such as injection current J, feedback strength κ, external cavity L, the LD will undergo from steady state, P1 state, period-doubling state, and chaos state. At steady state, there is only one optical frequency component emitted by a LD. However, as the laser experiences the Hopf bifurcation and enters the P1 state, a second dominant optical frequency component will appear. This is how the dual-frequency is generated in a LD with EOF. Our analysis shows that the dual-frequency varies with the external cavity length. This provides the flexibility for users to generate the desired beat frequency signals to suit practical needs. By using the proposed dual-frequency source, a novel Doppler LiDAR is presented for velocity measurement. The proposed design combines the merits of both the EOF system and DFDL, which have the advantages of low structural complexity, low power consumption, low cost, and light weight. Therefore, it has the potential to provide LiDAR measurement with high sensing resolution and a compact configuration for several practice usage, like in airborne and space-borne applications for velocity measurement. However, the beat frequency $f_{beat}$ generated using the system presented in this paper contains some noise caused by sidebands around the dual frequency components. To improve the measurement performance of the proposed system, some methods need to be explored to achieve narrower line width.

## Figures and Tables

**Table 1:** Physical meanings of symbols in Lang and Kobayashi (L-K) equations [33].

	Symbol	Physical Meaning	Value
LD Internal Parameters	$G_{N}$	Model gain coefficient	$8.1\times 10^{-13}\;m^{3}s^{-1}$
	$N_{0}$	Carrier density at transparency	$1.1\times 10^{24}\;m^{-3}$
	ε	Nonlinear gain compression coefficient	$2.5\times 10^{-23}\;m^{3}$
	Γ	Confinement factor	0.3
	$\tau_{p}$	Photon lifetime	$2.0\times 10^{-12}\;s$
	$\tau_{s}$	Carrier lifetime	$2.0\times 10^{-9}\;s$
	$\tau_{in}$	Internal cavity round-trip time	$8.0\times 10^{-12}\;s$
	e	Elementary charge	$1.6\times 10^{-19}\;C$
	V	Volume of the active region	$1\times 10^{-16}\;m^{3}$
	$\omega_{0}$	Unperturbed optical angular frequency of a laser diode, $\omega_{0}=2\pi c/\lambda_{0}$, where c is the speed of light, $\lambda_{0}$ is the wavelength of the LD	
	α	Line-width enhancement factor	
LD Controllable Parameters	J	Injection current	
	κ	Feedback strength	
	L	External cavity length	
	τ	External cavity round trip time, τ=2L/c	

**Table 2:** Comparison between our DFDL system and existing dual-frequency methods.

	This Work	[35]	[14]	[36]	[37]	[16]
Velocity measurement resolution	4.8 μm/s	26 μm/s	310 μm/s	327.9 μm/s	$7.5\times 10^{4}\;\mu m/s$	$1.2\times 10^{6}\;\mu m/s$
